# Metabolic engineering for the biosynthesis of bis-indolylquinone terrequinone A in *Escherichia coli* from L-tryptophan and prenol

**DOI:** 10.1186/s13068-023-02284-5

**Published:** 2023-03-02

**Authors:** Lijuan Wang, Yongdong Deng, Rihe Peng, Jianjie Gao, Zhenjun Li, Wenhui Zhang, Jing Xu, Bo Wang, Yu Wang, Hongjuan Han, Xiaoyan Fu, Yongsheng Tian, Quanhong Yao

**Affiliations:** 1 Shanghai Key Laboratory of Agricultural Genetics and Breeding, Biotechnology Research Institute of Shanghai Academy of Agricultural Sciences, 2901 Beidi Road, Shanghai, China; 2 Key Laboratory for Safety Assessment (Environment) of Agricultural Genetically Modified Organisms, Ministry of Agriculture and Rural Affairs, Shanghai, China

**Keywords:** Biosynthesis, Bis-indolylquinone, Terrequinone A, Dimethylallyl diphosphate (DMAPP), L-tryptophan (L-Trp), *Escherichia coli*

## Abstract

**Background:**

Terrequinone A is a bis-indolylquinone natural product with antitumor activity. Due to its unique asymmetric quinone core structure and multiple functional groups, biosynthesis is more efficient and environmentally friendly than traditional chemical synthesis. Currently, most bis-indolylquinones are obtained by direct extraction from fungi or by chemical synthesis. By focusing on the biosynthesis of terrequinone A, we hope to explore the way to synthesize bis-indolylquinones de novo using *Escherichia coli* as a cell factory.

**Results:**

In this study, a terrequinone A synthesis pathway containing the *tdiA*–*tdiE* genes was constructed into *Escherichia coli* and activated by a phosphopantetheinyl transferase gene *sfp*, enabling the strain to synthesize 1.54 mg/L of terrequinone A. Subsequently, a two-step isopentenol utilization pathway was introduced to enhance the supply of endogenous dimethylallyl diphosphate (DMAPP) in *E. coli*, increasing the level of terrequinone A to 20.1 mg/L. By adjusting the L-tryptophan (L-Trp)/prenol ratio, the major product could be changed from ochrindole D to terrequinone A, and the content of terrequinone A reached the highest 106.3 mg/L under the optimized culture conditions. Metabolic analysis of L-Trp indicated that the conversion of large amounts of L-Trp to indole was an important factor preventing the further improvement of terrequinone A yield.

**Conclusions:**

A comprehensive approach was adopted and terrequinone A was successfully synthesized from low-cost L-Trp and prenol in *E. coli*. This study provides a metabolic engineering strategy for the efficient synthesis of terrequinone A and other similar bis-indolylquinones with asymmetric quinone cores. In addition, this is the first report on the de novo biosyhthesis of terrequinone A in an engineered strain.

**Supplementary Information:**

The online version contains supplementary material available at 10.1186/s13068-023-02284-5.

## Introduction

Bis-indolylquinones are a kind of fungal natural products with broad pharmacological properties. Since the first discovery of Cochliodinol [1], the family of bis-indolylquinones has been expanding, with three new asterriquinones I–K isolated from the sponge-derived fungus *Aspergillus* recently [2]. All share a universal precursor–didemethylasterriquinone D (DDAQ D), and the diverse prenyl modifications on the indole group or benzoquinone core allow the bis-indolylquinones to present different biological activities, such as antiretroviral, antidiabetes, or antitumorigenic activities [3, 4]. Over the years, several bis-indolylquinones have been synthesized by synthetic chemists and pharmacologists [5, 6].

Among the many known bis-indolylquinones, terrequinone A isolated from *Aspergillus terreus* [7] attracted our attention because of its unique asymmetric quinone core. After 28 days of incubation, it could reach a yield of 1.11 mg/L in *A. terreus* broth [7]. Since it is a natural product with a complex structure and multiple functional groups, biosynthesis offers an innate advantage for the synthesis of this compound. Schneider et al. [4] proposed a one-pot, two-enzyme chemoenzymatic route for the synthesis of DDAQ D, a precursor of terrequinone A. Balibar et al. [15] overexpressed and studied the five genes *tdiA*–*tdiE*, respectively. In the presence of substrates and multiple cofactors, 100 μM of DDAQ D was successfully converted into terrequinone A through a step-by-step enzymatic reaction in vitro. However, the enzyme-catalyzed synthesis in vitro requires the external addition of expensive cofactors and cofactor regeneration systems. In addition, the product catalyzed by the previous enzyme in a biocatalytic system is often the substrate for the next enzyme. The delivery of substrates and products is usually space-limited, which reduces the efficiency of product synthesis [8]. In recent years, with the development of synthetic biology and metabolic engineering, the biosynthesis of high-value natural products in microorganisms has emerged as a promising route to perfectly avoid the above two problems. A variety of natural products, such as the alkaloid (*S*)-reticuline, the polyketide erythromycin, terpenoids nerol, taxadiene and artemisinin, have been successfully synthesized using *E. coli* as cell factories [9–13]. However, to the best of our knowledge, there is no report of terrequinone A synthesis in vivo using engineered bacteria so far.

The specific pathway for terrequinone A biosynthesis and modification in microorganisms has been elucidated: the first step is the conversion of L-tryptophan (L-Trp) to indole pyruvic acid (IPA) using the pyridoxal-5’-phosphate-dependent aminotransferase TdiD. Then, under the action of the monomodular nonribosomal peptide synthetase (NRPS) TdiA, two molecules of the IPA monomer are dimerized to form DDAQ D. Next, the oxidoreductase TdiC plays a role in reducing the keto group of the quinone core. Finally, the indole prenyltransferase TdiB and the chaperone TdiE participate in the first prenyl modification on the quinine core rather than the indole moiety by some unknown mechanism, resulting in the unique asymmetric quinine structure of terrequinone A. In contrast, the second prenylation reaction occurring on the indole moiety is performed by TdiB independently [14, 15]. This synthetic pathway involves three substances with different biological activities: DDAQ D, a universal precursor of bis-indolylquinones with anti-HIV activity [16], ochrindole D, a monoprenylated product with anti-insect activity [17], and terrequinone A, a diprenylated product with antitumor activity [7]. The terrequinone A synthesis pathway is typical of those that exhibit different biological activities through successive prenyl modifications. Therefore, this pathway was reconstituted in *E. coli* in this study.

In the biosynthetic pathway of terrequinone A, dimethylallyl diphosphate (DMAPP) is a major substrate, and its production in *E. coli* is dependent on the methylerythritol 4-phosphate pathway (MEP pathway), a glucose metabolite-based pathway that requires seven reactions to obtain DMAPP/isopentenyl pyrophosphate (IPP) [18]. Due to the close relationship between the MEP pathway and central carbon metabolism pathways and the complex regulatory mechanism [19], many metabolic engineering modifications based on the MEP pathway to enhance DMAPP production have not achieved the desired effect [20]. Some studies have adopted heterologous expression of the mevalonate pathway (MVA pathway) to enhance the supply of IPP/DMAPP, which has achieved good results, but the synthesis steps are still complicated [21, 22]. Recently, a two-step enzymatic pathway (isopentenol utilization pathway, IU pathway) has been creatively proposed for biological DMAPP/IPP production. This pathway takes exogenous isoprenol or prenol as substrate, which is separated from central carbon metabolism, thus making it possible to maintain high throughput. The approach effectively addresses the lack of DMAPP/IPP supply in isoprenoid biosynthesis, which has been the major focus for the past 20 years [20].

In this study, an engineered strain for terrequinone A production was constructed by introducing the *tdiA*–*tdiE* genes from *A. nidulans*, *sfp* from *Bacillus subtilis* and a two-step DMAPP synthesis system into *E. coli*. The strain could successfully produce terrequinone A using low-cost L-Trp and prenol as substrates. The culture conditions of engineered strain were also optimized. This study provides a metabolic engineering strategy and method for the synthesis of terrequinone A and other similar bis-indolylquinones with asymmetric quinone cores.

## Results and discussion

### Construction of multi-monocistronic expression vectors

**Fig. 1:**
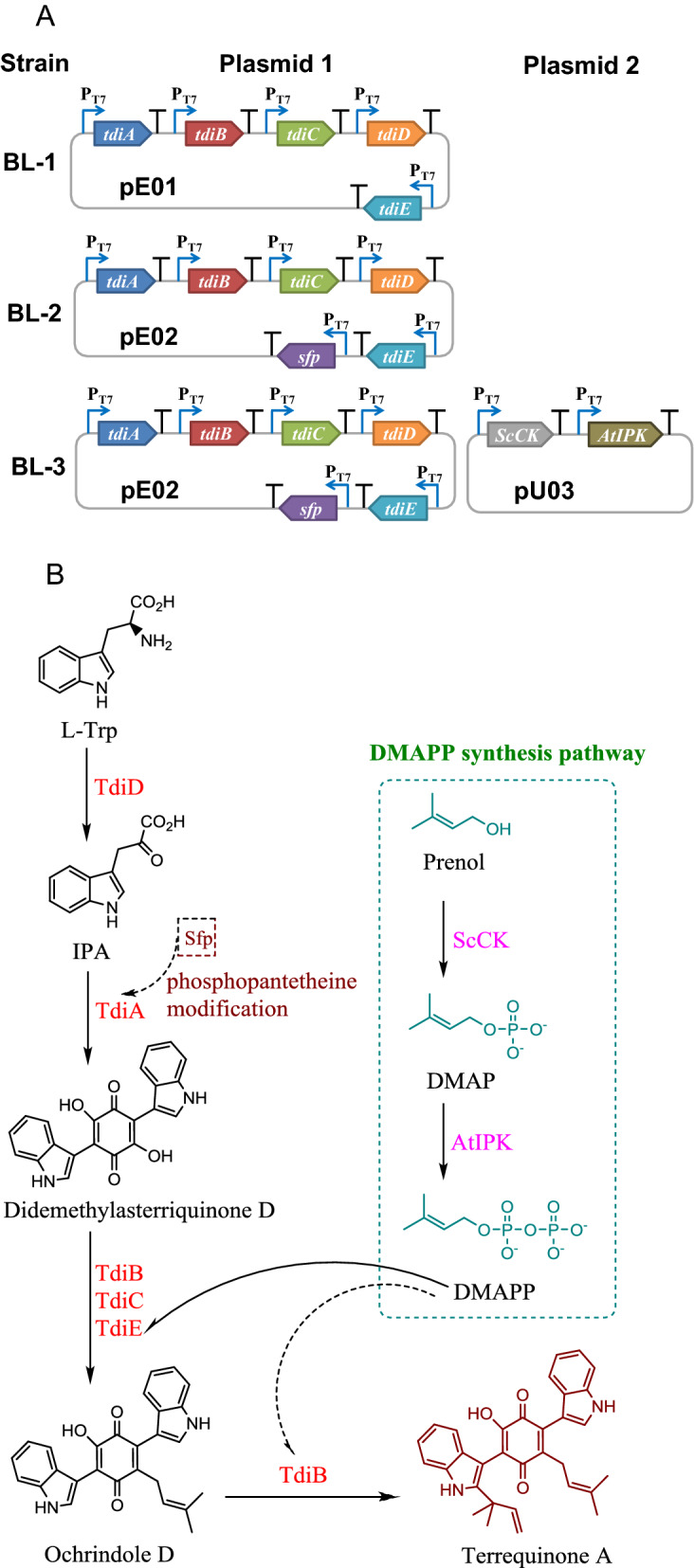
Biosynthesis of terrequinone A in *E. coli*. **A** The schematic representation of multi-monocistronic expression vectors **B** Engineered pathway for the terrequinone A synthesis in *E. coli*. *TdiD* L-Trp aminotransferase, *TdiA* monomodular nonribosomal peptide synthetase, *TdiB* indole prenyltransferase, *TdiC* oxidoreductase, *TdiE* chaperone, *Sfp* phosphopantetheinyl transferase, *ScCK* choline kinase, *AtIPK* isopentenyl phosphate kinase

To actively express genes from different sources in *E. coli*, all selected genes *tdiA*–*tdiE*, *sfp*, *ScCK* and *AtIPK* were optimized (Additional file 1: Table S1). Among them, *tdiA*–*tdiE* and *sfp* genes constituted the terrequinone A synthesis module, and *ScCK* and *AtIPK* genes constituted the DMAPP synthesis module. In addition, the coordinated expression of multiple genes should be considered in the construction of synthetic biological pathways. In the traditional multi-gene construction approaches, the expression of genes located near the 5' end of the operon is higher than that of genes located near the 3' end, which is not conducive to the balanced regulation of metabolic pathways [23]. In recent years, multi-monocistronic vectors have been developed, in which each gene may contain its own promoter and terminator [24, 25]. Such vectors show an obvious advantage in gene coordinated expression, as this design is able to reduce the premature transcription termination and mRNA degradation [26]. Hence, similar vectors that connected the T7 promoter and terminator sequences at both ends of each gene sequence were performed in this study (Table 1, Fig. 1A). The successful construction of the engineered strains harboring the heterogeneous genes was verified by PCR (Additional file 1: Figure S1) and DNA sequencing.

**Table 1:** Plasmids and strains used in the present study

Designation	Genotype or description	Source
*Plasmids*		
pET-28a ( +)	Kan^R^, P_T7_	Novagen
pUC19	Amp^R^, P_T7_	Novagen
pE01	pET-28a ( +) origin, Kan^R^, P_T7_, *tdiA*–*tdiE*	This study
pE02	pET-28a ( +) origin, Kan^R^, P_T7_, *tdiA*–*tdiE* and* sfp*	This study
pU03	pUC19 origin, Amp^R^, P_T7_, *ScCK* and *AtIPK*	This study
*Strains*		
BL21-AI	F-ompT gal dcm lon hsdSB(rB^−^ mB^−^) araB::T7RNAP-tetA	Novagen
BL-control	BL21-AI/pET-28a ( +)	This study
BL-1	BL21-AI/pE01	This study
BL-2	BL21-AI/pE02	This study
BL-3	BL21-AI/pE02/pU03	This study

### Construction of the terrequinone A synthetic pathway in* E. coli*

**Fig. 2:**
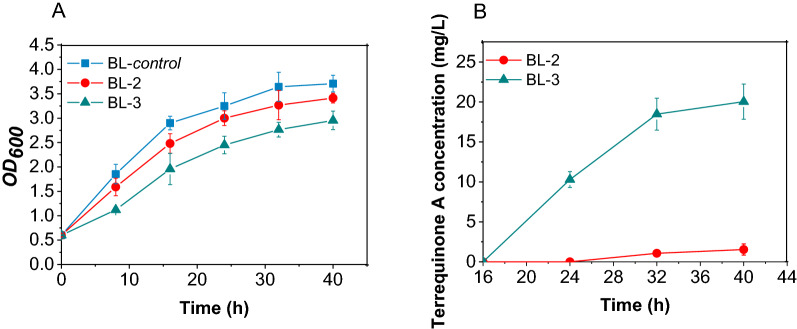
Time profiles of **A** biomass (OD_600_) and **B** the terrequinone A concentration in the cultivation of different strains at 30 ℃

The engineered strain BL-1 harboring *tdiA*–*tdiE* was constructed by transferring the multi-monocistronic vector pE01 into BL21-AI (Table 1). As a result, terrequinone A could not be detected in the fermentation broth of strain BL-1. TdiA, an NRPS with A-T-TE tridomain, requires its T domain to be activated from apo form to holo form through phosphoantenthienyl transferase to realize the function [15]. However, this post-modification of TdiA is not present in *E. coli*. Therefore, the promiscuous phosphopantetheinyl transferase gene *sfp* from *B. subtilis* [27] was introduced into *E. coli* to functionally reconstitute the terrequinone A synthesis pathway. As expected, TdiA was activated by Sfp, and by adding 0.5 g/L of L-Trp, BL-2 harboring *tdiA*–*tdiE* and *sfp* genes produced 1.54 mg/L of terrequinone A after 40 h of cultivation at 30 ℃ (Fig. 2B), indicating that the terrequinone A synthetic pathway was successfully constructed in *E. coli*. Although *E. coli* itself produced trace amounts of L-Trp, terrequinone A could not be detected in the culture of BL-2 without the supplement of L-Trp.

### Effect of enhancing DMAPP synthesis pathway on terrequinone A production

DMAPP is an important substrate for prenyl modification of bis-indolylquinones. The synthesis of one molecule of terrequinone A requires two molecules of DMAPP (Fig. 1B), and the DMAPP produced by the MEP pathway in *E. coli* is far from meeting the requirements of terrequinone A synthesis, which becomes the bottleneck for terrequinone A production by strain BL-2. The addition of exogenous DMAPP is not only expensive, but also cannot be effectively utilized due to the barrier of substrate transfer by cytoderm. To increase the supply of endogenous DMAPP, the IU pathway containing the *ScCK* and *AtIPK* genes [20] was also overexpressed in strain BL-3. The total engineered pathway for the terrequinone A synthesis is shown in Fig. 1B.

In the presence of 0.5 g/L L-Trp and 0.21 g/L prenol (an L-Trp/prenol molar ratio of 1:1), two main peaks at 8.43 min and 8.94 min appeared in the total ion chromatogram (TIC) of the chloroform extract of BL-3 culture (Additional file 1: Figure S2A). The MS signals for both were 423.2 [M + H]^+^ and 491.3 [M + H]^+^ (Additional file 1: Figures S2B and C), respectively, which matched the MS data for ochrindole D and terrequinone A measured by Balibar et al. [15]. Thus, terrequinone A synthesis pathway and IU pathway were functionally constructed in BL-3, and the strain BL-3 produced 20.1 mg/L of terrequinone A after 40 h of cultivation at 30 ℃ (Fig. 2B). The yield of terrequinone A synthesized by the engineered strain was increased by approximately 12-fold through the introduction of DMAPP enhanced synthesis pathway. In addition, although BL21-AI was used as a host to reduce the protein toxicity to cells, as shown in Fig. 2A, during the protein induction period (0–16 h), the expression of heterologous multi-gene still had a significant inhibitory effect on bacterial growth. This emphasized the importance of minimizing the number of heterologous genes in artificially constructed pathways. The IU pathway achieved DMAPP production with only two genes, which is the simplest pathway for DMAPP synthesis reported so far. The introduction of this pathway greatly eased the stress of protein expression on cells and enabled BL-3 to produce terrequinone A directly from low-cost substrates L-Trp and prenol.

### Optimization of culture conditions for terrequinone A production

**Fig. 3:**
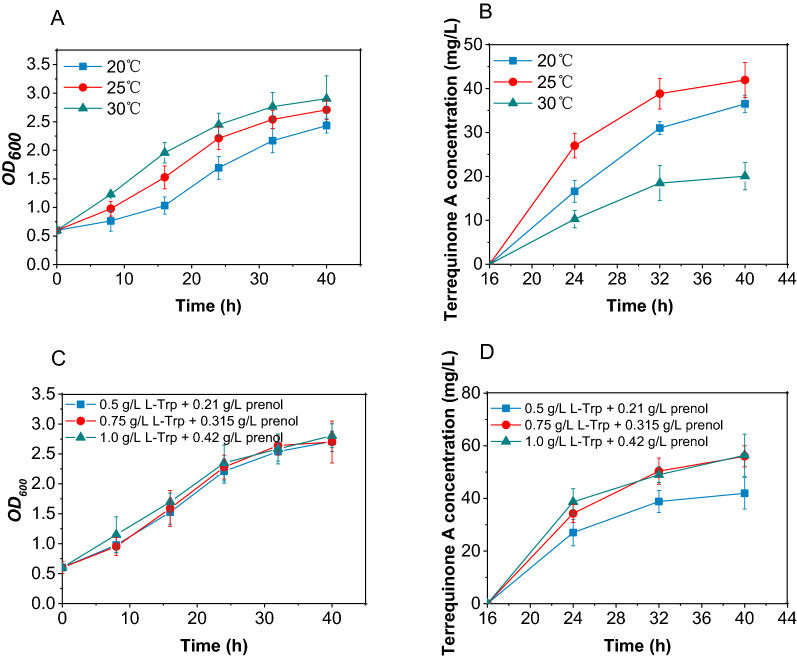
Optimization of culture conditions for terrequinone A synthesis by BL-3. Effect of induction temperature on **A** biomass (OD_600_) and **B** terrequinone A production. Effect of substrate concentration on **C** biomass (OD_600_) and **D** terrequinone A production. The protein induction period (0–16 h), the terrequinone A synthesis period (16–40 h)

Temperature can effectively affect exogenous protein activity in engineered bacteria by regulating protein expression. In the study, the induction temperature was optimized to improve the production of terrequinone A. To reduce the consumption of L-Trp in other cellular metabolisms and the volatilization of prenol, the two substrates were added at 16 h after the protein induction period, when proteins in the terrequinone A synthesis pathway were fully expressed. As shown in Fig. 3A, the cell density was positively correlated with the induction temperature. Figure 3B shows that the induction temperature had a great influence on the product synthesis. The lowest yield was obtained at 30 ℃ induction despite the fastest cell growth. While the highest amount of terrequinone A was produced at 25 ℃ induction, indicating that inducing at 25 ℃ was more favorable for active protein expression, thus achieving higher yield.

Under an L-Trp/prenol molar ratio of 1:1, effect of substrate concentration on the synthesis of terrequinone A was investigated. As shown in Fig. 3C, the concentration of substrates had little effect on cell growth density. The addition of 0.75 g/L L-Trp and 0.315 g/L prenol (or 1.0 g/L L-Trp and 0.42 g/L prenol) increased the yield of terrequinone A to approximately 56 mg/L (Fig. 3D). Finally, 0.75 g/L L-Trp and 0.315 g/L prenol were chosen for the terrequinone A synthesis for economic consideration.

### Changing the major prenylated product by adjusting the L-Trp/prenol ratio

**Fig. 4:**
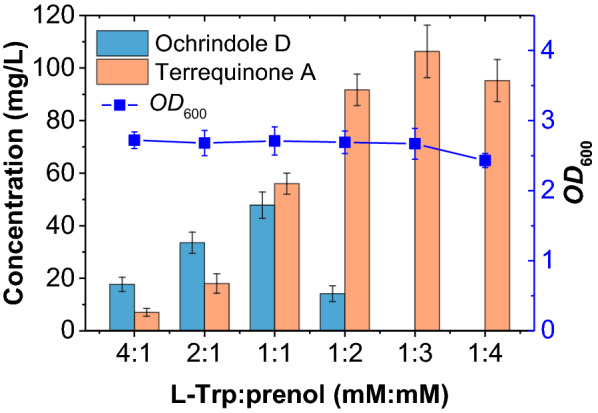
Changes in the prenylated products of BL-3 under different L-Trp/prenol ratios

Under an L-Trp/prenol molar ratio of 1:1, a considerable amount of ochrindole D (monoprenylated DDAQ D) was produced along with terrequinone A (diprenylated DDAQ D) by strain BL-3. Therefore, the synthesis of terrequinone A at different substrate ratios was also investigated. As shown in Fig. 4, with the increase of prenol, the major product changed from ochrindole D to terrequinone A until ochrindole D was completely converted to terrequinone A. The content of terrequinone A reached the highest 106.3 mg/L when the molar ratio of the substrates was 1:3. However, the level of terrequinone A decreased when the ratio was 1:4, along with a slight decrease in cellular *OD*_600_ value. This may be due to the toxicity of both short-chain-alcohol prenol and the intermediate product DMAPP to the cells [28, 29]. Whether the metabolic strength of the upstream and downstream modules of the synthesis pathway is balanced severely affects the final yield of the target product. If the upstream module is strong and the downstream module is weak, it tends to cause excessive accumulation of intermediate metabolites, which will not only reduce the carbon utilization rate but also may cause cytotoxicity and affect the growth of microorganisms, leading to a decrease in yield. In this study, the IU pathway separated from the central carbon metabolism was introduced into *E. coli* to enhance the DMAPP production. Therefore, it is possible to balance their metabolic intensity by adjusting the ratio of the substrates of the two synthesis modules, and a better synthesis effect can be achieved.

### L-Trp metabolic analysis

**Fig. 5:**
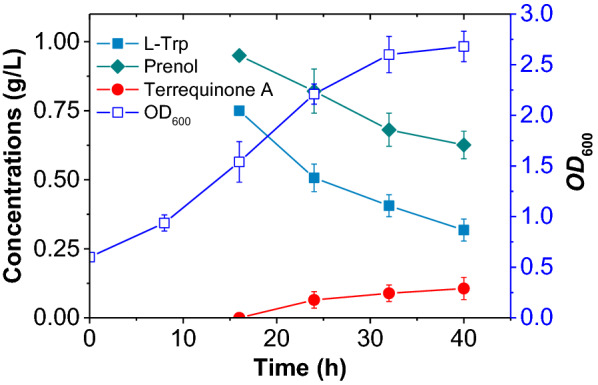
Terrequinone A biosynthesis in BL-3 under the optimized culture conditions

**Fig. 6:**
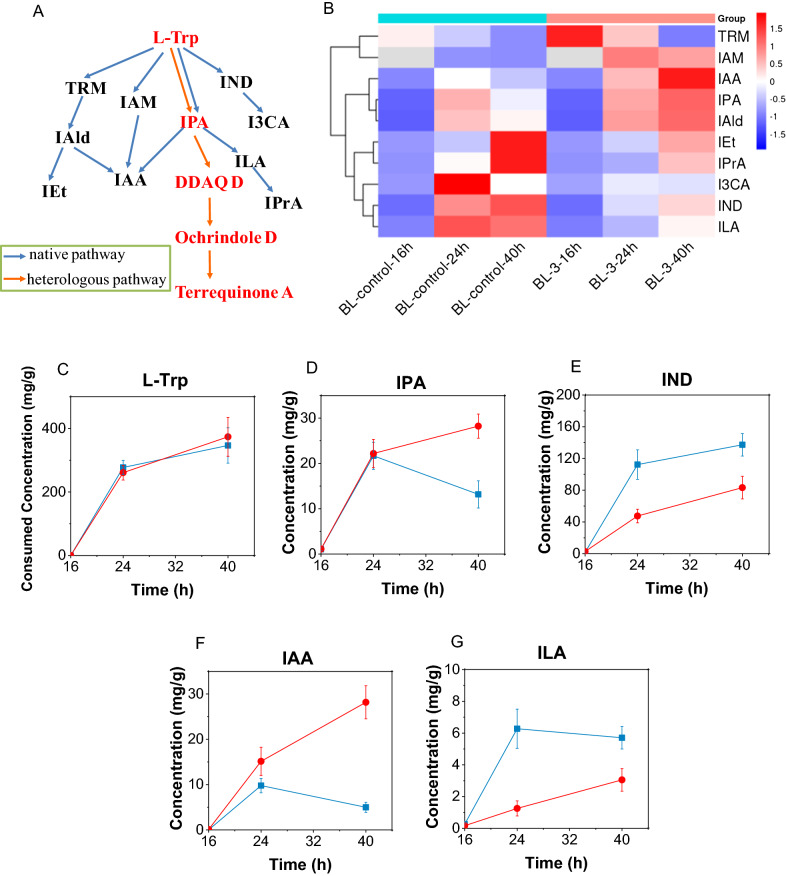
Effect of heterologous pathway on L-Trp metabolism. *L-Trp* L-Tryptophan, *IPA* indole pyruvic acid, *IND* Indole, *I3CA* indole-3-carboxylic acid, *TRM* tryptamine, *IAM* indole-3-acetamide, *IAld* indole-3-aldehyde, *IAA* indole-3-acetic acid, *IEt* tryptophol, *ILA* indole-3-lactic acid, *IPrA* indole-3-propionic acid, *DDAQ D* Didemethylasterriquinone D. Concentrations of L-Trp consumed and L-Trp catabolites produced were normalized to dry cell weight. **A** L-Trp degradation pathway in *E. coli*. **B** Heatmap of L-Trp catabolites with the addition of 0.75 g/L L-Trp and at 16 h. The content comparison of **C** L-Trp, the key catabolites **D** IPA, **E** IND, **F** IAA, and **G** ILA between () BL-control and () BL-3

Under the optimized conditions, the growth curves as well as the substrate consumption during the terrequinone A synthesis were also examined simultaneously. As shown in Fig. 5, despite the addition of the substrates L-Trp and prenol at 16 h, they were still consumed in large amounts, and the yield of terrequinone A was relatively low. In particular, with the multiple functional groups such as carboxyl, amino and indole groups, L-Trp can be modified to form a variety of different structural units and is an important substrate for many natural products [30], and L-Trp also participates in various metabolisms of *E. coli* as a nutrient. Therefore, it is important to clarify the metabolic flow of L-Trp in recombinant bacteria to improve the biosynthetic yield of active products with L-Trp as a substrate. In this study, several L-Trp metabolic pathways (Fig. 6A) were detected in strains BL-control and BL-3 by UHPLC–MS, and L-Trp and its 10 metabolites were quantified. As shown in Fig. 6B–G, the introduction of heterologous terrequinone A synthesis pathway significantly changed the metabolic flow of L-Trp, and a considerable fraction of L-Trp was used for terrequinone A synthesis in strain BL-3. Indole (IND) and indole-3-acetic acid (IAA) were down-regulated and indole-3-lactic acid (ILA) was up-regulated among several key L-Trp catabolites. The metabolic pathway from L-Trp to IPA was also present in BL-control, and the concentration of IPA in BL-3 was almost the same compared with the control at the initial phase of terrequinone A synthesis, indicating that the activities of TdiD and TdiA were in a dynamic equilibrium. Subsequently, a decrease in TdiA activity resulted in the accumulation and up-regulation of IPA.

Although TdiD enhanced the conversion of L-Trp to IPA in BL-3, the consumption of L-Trp did not increase obviously compared to BL-control (Fig. 6C). This may be due to more degradation of L-Trp to indole in BL-control than in BL-3 (Fig. 6E). In *E. coli* MG1655, the catabolism of L-Trp mainly depends on the tryptophanase encoded by the tnaA gene, which catalyzes the conversion of L-Trp to indole and pyruvate [31]. Knockdown of the *tnaA* gene in the strain could effectively prevent the flow of L-Trp into the indole metabolism branch and reduce the intracellular degradation of L-Trp [32], and this metabolic pathway modification could be attempted to further improve the terrequinone A yield in the future.

## Conclusions

In this study, an engineered strain for terrequinone A production was successfully constructed by integrating a terrequinone A synthesis module and a two-step enzymatic DMAPP synthesis module in *E. coli*. A comprehensive engineering approach was adopted to synthesize terrequinone A and enhance its production, including optimization of gene nucleotide sequences, construction of modular pathways via multi-monocistronic expression vectors, enhancement of the DMAPP synthesis pathway, optimization of culture conditions, and adjustment of the substrate ratio of the two synthesis modules. The engineered strain eventually produced 106.3 mg/L terrequinone A from low-cost L-Trp and prenol. Metabolic analysis of L-Trp demonstrated that a large amount of L-Trp flowed to the indole metabolism branch during the synthesis of terrequinone A in BL-3, and the knockdown of *tnaA* gene could be tried to further improve the terrequinone A yield in the future. This study provided a metabolic engineering strategy for the efficient synthesis of terrequinone A. It is also expected to synthesize more new bis-indolylquinones with an asymmetric quinone core by mining suitable prenyltransferases in Genbank to replace TdiB in the engineered pathway.

## Materials and methods

### Chemicals, plasmids and strains

All chemicals used in the study were purchased from J&K Scientific Ltd. (Beijing, China). Reagents for molecular biology experiments were purchased from TaKaRa Biotechnology (Dalian) Co., Ltd.

The gene cluster *tdiA*–*tdiE* (GenBank No. EF550581.1, EF550582.1, EF550583.1, EF550584.1 and EF550585.1) for the synthesis of terrequinone A from *A. nidulans*, *sfp* (GenBank No. X65610.1) from *B. subtilis*, *ScCK* (GenBank No. AAA34499.1) from *Saccharomyces cerevisiae* and *AtIPK* (GenBank No. AY150412.1) from *Arabidopsis thaliana* were optimized to improve the probability of gene activity expression. The optimized gene sequences are listed in Additional file 1: Table S1. The T7 promoter and terminator (Additional file 1: Table S1) were designed to the 5' and 3' ends of each gene, respectively, to construct the multi-gene expression cassettes *tdiABCDE*, *tdiABCDE-sfp* and *ScCK-AtIPK*. These expression cassettes were chemically synthesized by GenScript Biotech Co. (Nanjing, China) and double-digested with *EcoR*I and *Hin*dIII, and then inserted into pET-28a ( +) or pUC19 to generate plasmids pE01, pE02 and pU03, respectively. The plasmids and strains used in this study are listed in Table 1. The successful construction of the vectors carrying exogenous genes was verified by PCR and DNA sequencing. PCR was performed using plasmids extracted from BL-1, BL-2 and BL-3 as templates. The specific Primers used for the PCR are listed in Additional file 1: Table S2.

### Culture conditions

The single colonies were selected and cultured overnight at 37℃ in LB medium (10 g tryptone, 5 g yeast extract and 10 g NaCl per liter). The fresh seed culture was inoculated in a conical flask containing 50 mL LB medium and cultured at 220 rpm and 37 ℃. When *OD*_600_ reached 0.6, the cells were collected, washed with double-distilled water, and re-suspended with optimized M9 minimal medium (15 g glycerol, 6 g Na_2_HPO_4_, 3 g KH_2_PO_4_, 1 g NH_4_Cl, 0.5 g NaCl, 0.12 g MgSO_4_, 0.011 g CaCl_2_, 2.9 mg ZnSO_4_·7H_2_O, 0.2 mL 1% (w/v) vitamin B1 and 5 g acid-hydrolyzed casein per liter) of equal volume. Meanwhile, L-arabinose was added to the medium with a final concentration of 2 g/L to induce the expression of heterologous proteins, and cultivation was continued for a further 16 h. The induction temperature was set to 20, 25 or 30 ℃ according to the requirements of the experiment. After that, substrates L-trp and prenol with different concentrations were added and incubated at 30 ℃ for 24 h to synthesize ochrindole D and terrequinone A. Appropriate antibiotics (ampicillin, 100 mg/L; kanamycin 50 mg/L) were added to the medium following the resistance of the engineered bacteria. All experiments were performed in triplicate.

### Purification of ochrindole D and terrequinone A

Ochrindole D and terrequinone A were purified from two liters of fermentation broth for quantitative analysis. The cells were collected by centrifugation at 7000 rpm for 10 min, suspended in 200 mL distilled water and crushed with an ultrasonic oscillator (JY92-II, Scientz Biotech. Co., Ltd). The broken cell suspension was extracted twice with an equal volume of chloroform. The organic layer was evaporated under vacuum and then redissolved in 20 mL of methanol. Ochrindole D and terrequinone A in the extract were purified by HPLC (Agilent 1100) equipped with a semi-preparation column (Agilent ZORBAX SB-C18 column, 250 mm × 9.4 mm × 5 μm) and monitored at 280 nm. The mobile phase was a gradient of acetonitrile/water containing 0.1% TFA (from 5 to 100% in 20 min) at a flow rate of 4 mL/min.

### Analytical methods

The cell biomass was determined by absorbance at 600 nm (*OD*_600_) with a microplate reader (Tecan Infinite M200). Two hundred μl of fermentation broth was quenched with twice the volume of methanol, ultrasonicated for 10 min, and centrifuged at 10,000 rpm for 5 min. The concentrations of L-Trp, ochrindole D and terrequinone A in the supernatant were detected by HPLC with an ultraviolet spectrophotometric detector (Agilent 1100 VWD) at 280 nm and an Athena C18 reversed-phase column (250 mm × 4.6 mm × 5 μm, ANPEL Inc., China) at 35 ℃, using a gradient of acetonitrile/water containing 0.1% TFA (from 5 to 100% in 20 min) at a flow rate of 1 mL/min. The purified ochrindole D and terrequinone A mentioned above were prepared for the standard curve. Ochrindole D and terrequinone A were identified by LC–MS using a TSQ Quantum-Accela system with a Shim-pack GIST C18 column (150 mm × 2.1 mm, 3 μm, Shimadzu Co., Japan) and positive ion mode ESI. Solvent A: 0.1% (v/v) formic acid in H_2_O; solvent B: 0.1% (v/v) formic acid in acetonitrile, flow rate: 0.2 μL/min. The gradient was: initial hold in 20% B for 3 min, then to 90% B within 10 min. To detect the concentration of prenol in the samples, 200 μL of fermentation broth was extracted with an equal volume of ethyl acetate (isoamyl alcohol was added as the internal standard), and the organic phase was detected by a 7890B GC system (Agilent Technologies) equipped with a flame ionization detector and an HP-5 capillary column (30 m × 0.32 mm, 0.25 μm, Agilent). The oven temperature was initially held at 50 ℃ for 1 min, then increased to 100 ℃ at the rate of 5 ℃/min, and further increased to 150 ℃ at the rate of 25 ℃/min. The injector and detector were held at 250 ℃ and 270 ℃, respectively.

### L-Trp metabolism

The substrates L-trp and prenol were added at 16 h and the cultures of BL-control and BL-3 were sampled at 16 h, 24 h and 40 h, respectively. Ten ml of fermentation broth was ultrasonicated for 10 min and freeze-dried in vacuum to lyophilized powder. An appropriate amount of lyophilized powder was added to 100 μL of 80% methanol and oscillated for 60 s, and then 900 μL of 10% methanol was added to oscillate for 60 s. The extract was centrifuged at 10,000 rpm for 5 min to obtain the supernatant for detection. L-Trp catabolites were measured by UHPLC–MS proposed by Chen et al. [33] with slight modifications, using a Waters Acquity UPLC system equipped with an HSS T3 column (2.1 × 150 mm, 1.8 μm, Waters) and an API 5000 triple quadrupole instrument (AB Sciex). The mobile phase was composed of solvent A (0.1% formic acid in water) and solvent B (0.1% formic acid in methanol). The gradients were: 0 ~ 2 min, 1% B; 2 ~ 3 min, 1 ~ 30% B; 3 ~ 3.5 min, 30% B; 3.5 ~ 8 min, 3 ~ 50% B; 8 ~ 10 min, 50 ~ 95% B; 10 ~ 11 min, 95% B. The flow rate was 0.3 mL/min. Data analysis and graphics were performed through bioDeep™ data analysis platform (http://www.biodeep.cn/).

## Supplementary Information

**Additional file 1: Table S1.** The gene sequences involved in this study. **Table S2.** Primers used in this study. **Figure S1.** PCR amplified fragments using plasmids from engineered strains as templates. **Figure S2.** LC–MS analysis of the BL-3 culture after chloroform extraction.

## Data Availability

All data generated or analyzed during this study are included in this published article and its Additional information files.

## Abbreviations

L-Trp: L-tryptophan
DDAQ D: Didemethylasterriquinone D
IPA: Indole pyruvic acid
NRPS: Nonribosomal peptide synthetase
MEP pathway: Methylerythritol 4-phosphate pathway
MVA pathway: Mevalonate pathway
DMAPP: Dimethylallyl diphosphate
IU pathway: Isopentenol utilization pathway
IND: Indole
IAA: Indole-3-acetic acid
ILA: Indole-3-lactic acid
PPA: Phenylpyruvic acid
