# Supplementary material for: Metabolic engineering for the biosynthesis of bis-indolylquinone terrequinone A in Escherichia coli from L-tryptophan and prenol
Source: Biotechnol Biofuels Bioprod. 2023 Mar 2;16:34. doi: 10.1186/s13068-023-02284-5 (PMC9979454; doi:10.1186/s13068-023-02284-5)
Supplement: Supplementary file 1 — Additional file 1: Table S1. The gene sequences involved in this study. Table S2. Primers used in this study. Figure S1. PCR amplified fragments using plasmids from engineered strains as templates. Figure S2. LC–MS analysis of the BL-3 culture after chloroform extraction. [file 13068_2023_2284_MOESM1_ESM.docx]

Supplementary Information

For

**Metabolic engineering for the biosynthesis of bis-indolylquinone terrequinone A in *Escherichia coli* from L-tryptophan and prenol**

Lijuan Wang^1†^, Yongdong Deng ^1†^, Rihe Peng^1^, Jianjie Gao^1^, Zhenjun Li^1^, Wenhui Zhang^1^, Jing Xu^1^, Bo Wang^1^, Yu Wang^1^, Hongjuan Han^1^, Xiaoyan Fu^1^, Yongsheng Tian^1*^, Quanhong Yao^1*^

^†^ Lijuan Wang and Yongdong Deng have contributed equally to the article

*Correspondence: [tys810508@126.com](mailto:tys810508@126.com); [yaoquanhong_sh@aliyun.com](mailto:yaoquanhong_sh@aliyun.com)

**^1^** Shanghai Key Laboratory of Agricultural Genetics and Breeding, Biotechnology Research Institute of Shanghai Academy of Agricultural Sciences, 2901 Beidi Road, Shanghai, China

**Table S1.** The gene sequences involved in this study

Sequence design of vector pE01 expression region:

Sequence design of vector pE02 expression region:

Sequence design of vector pU03 expression region:

**T7 promoter**

CGATCCCGCGAAATTAATACGACTCACTATAGGGGAATTGTGAGCGGATAACAATTCCCCTCTAGAAATAATTTTGTTTAACTTTAAGAAGGAGATATA

**T7 terminator**

TAGCATAACCTTGGGGCCTCTAAACGGGTCTTGAGGGGTTTTTTG

***tdiA***

ATGGCACCATCTAAGACTGAAATTGCACCACTTAGAGCAGCAAAATATCCATTTGGTAACATTGTTGATGCTCTTCGTCATGCTGCTGCACATACCGATGAGGGTATCATCGTCTACCATCCAAACTCCATCTCCACCTCCTCCCCACCACAGACCGTCTCCTACAAAGACCTGCTGCATCAGGCTGAGGCAAACGCAACTCGTCTGTTGCAGCAGAAGCTGTGTTCTCCAAAGTCCATCGTCCTGGTTCACTTCGAGTCTGCACTGGACTCCATCGTCTGGTACTGGTCTGTCCTTCTGGCTGGTGGTATTCCTGCACTGACTGGTCCTGGTATGTTCTCTCAGAACCCTGCTGATCGTGAACGTCATCTGCGTCATCTGTCTGAGACCCTGAACTCTCCTGTCTGTCTGACTCGTCCTGCACTGTTGGCACCATTTGAAGAGCAGACTGCTGATGACCGTATCAAAGCTCGTACTGTCGATGAGATCCTGGCTGCACCTGAAATCGCTGATGTTGCTGACGCACCACTGCCTGCACTGACTCCATCTTCTACTGACATGCTTGCACTGATGCTGACTTCTGGTTCTTCTGGTAATGCCAAGGCTGTTCCACTGACTCATCAACAGCTGCTTGCTGCCTTTCGTGGTAAGTCTACTGCTGCTTCTCTGCGTTTCCCACGTTCTCCTTTCCTGTCTTGGGTTCACATGGATCATGTTGCTAACCTGGTTCATTGCCACATCTTCGCTATCGTTTCTGGTATCTCCCAGATCCAAGTTCCTGCACCTGACCTGCTGATCAACCCTGCACAGCTTCTGAACCTGATCTCTCGTCACCGTGTCTCTCGTACCTTCATGCCAAACTTCCTGTGTGCCAAGTTGCGTCGTCAGCTGGAGTCTGGTTCTCCAGAGTACATCCTTGACCCTGGTCTGAACCTGGAAACCCTGTACATCGATACTGGTGGTGAGGCTAACGTCACTGAGGTCTGCATTGCACTGCAATCTCTGCTGTCTCGTTACGGTGCTCCTGACAACGTCTTCAAGCCATCCTTCGGTATGACTGAGACTGTTGCTGGTTGCATCTTCAACTCTCATTGTCCATCCTATGACCATGCACAACGTCATGAGTTCGCATGTCTTGGTAAGCCTATGCCTGGTGTTCGTATGCGTGTCACTCGTCTGGATACTCCATCTGAAGAGGCTGCTCCTGGTGAACGTGGTTCTCTGGAAGTCACTGGTGAAGTTGTCTTCAAGGGTTACTACAACAACCCTGCTGCAACTGCTGAGGCATTCACCTCTGATGGTTGGTTCCGTACTGGTGATCTGGCATTCATTGACTCCAATGGTAATCTGCATCTTGATGGTCGTACTAAGGAGATGATCAACATCAATGGTGTCAAATACCTGCCATACGAACTGGATGCTGCTCTTGAGCAGGCACAGATTCCTGGTGCAACTCCATCTTACTTCTGCACCTTCTCCTCTCGTGATGCAACTATGGACACTGAGGTTGTCGTTGTCCTGTACCTTCCATCCTACGTCGAGTCTGATGATGAAGCACGTTTCTCTACTCAATCCTCTATCATCCGTGTTGTTGCAATGCATACTCGTTCTCGTCCACGTGTCGTTCCACTGCGTCCACAGGACATGCCAAAGTCCACTCTGGGTAAGCTGTCTCGTGCAAAGCTGAAGACTGCACTGGAAGAAGGTCAGTTCGCAACTCAACAACAAATCAACGACGAAGCCATCCGTCGTTATCAGCAGAAGACTCGTGCATCTCCTGAAACTCCTGACGAAGCTGTCATTCTGGATATTATCAAGGAACAGCTGGAGATCCGGTCTGATGATGACAGCTTTGGCGTCAACGATTCAATCCTGTCCATCGGTGCAACCTCTATGGATCTGGTTGCAATCATTCACCGTATCAACAAGTGTCTTCAGCCATCTCAGCCACTGCGTCTGACTGACATCCTGAAAGACTCCACTGCTCGTGGTCTGGCTGTTGCACTTGCAACTGGTGCTGCACCACGTTCTCAGGATCAGTCTTCTACTCATGTCTACGATCCTGTTGTCACTCTTCAACCACATGGTACTAAGTCTCCACTGTGGCTGGTTCATCCTGGTGTTGGTGAGGTCCTTGTCTTCGTCAACCTTGCACACCACATCACCGACCGTCCTGTCTACGCATTCCGTGCAAAGGGTTTCAATGCTGCTGCTGGTCTGCCTGAGACTCCATTCACCTCTCTTGAGGAGTTGTTCACTACCTACCGTGATGCAATCAAGGAACGTCAACCACATGGTCCATACGCAATCGCTGGTTACTCCTTCGGTGGTATGGTTGCATTCGAAGTCTCCAAGCTGCTTGAACAGGATGGTGATGAGGTTCGTTACTGTGGTTCTTGGAACCTTCCACCACACATCAAGTTCCGTATGCGTGAACTCGTCTGGGAGGAATGCGTTATCCATCTATTCTACTTCGTTGGTCTGATGACTGAACTGGCTGCATACACTCACAAACCAACTCTTCAGGAGTTCAACCGTGCAAACCGTCGTCTGGATGCTATCCGTTACCTGCGTCAACACTGTGATGCTGCACGTTGGGATGAACTTGGTCTGTCTGAGGAGTACTATCTGCTGTGGGTTGGTCTGGCATCTAACATGCAATCCCTTGCTGTTGACTACGAACCATCTGGTTCCGTCAAGTGCATGGATGTCTTTGTTGCTGATCCATTGTCTCATGTTGCAAAGGACCGTATCGACTGGGTTGAGGGTCGTTTGTCTGCATGGAAGGAGTTCGTTCGTGAGGATGTCCGTTTCCATGATGTCCAAGGTGCACACTACACCATGCTGAACCGTGAGTACGTCGAGGCATTCGCTGGTACTCTGAAGAACGTCCTGCGTGAGCGTGGTCTGTAA

***tdiB***

ATGGCTACTGAATACTGGTCTCGTCATCTTCGTTCTGTTCTTGCACCACTGTTCGCTGCTGCTGGTACTTACTCTCCTGAAGATCAGGAGTCTCATCTGGCCTTCATTGACGAGCACATTGCACCAAACCTGGGTCCACTGCCTTGGGAACCACATGGTCCATACTCTACTCCATCTTCTCTTGTTGGTTCTCCATTCGATCCATCTATCAACATCGTCTCTTCTGGTAAGGCCAAGGTTCGTTTCGACTTCGATGTCATCTCTCCACCTGATCGTACTGGTCCTGACCCATTCGCTGAGGGATCTGCTCGTGAGATCCTTCATCGTCTTGCTGACCTTGTTGGTGCTGACACTCAGTGGATGGGTTATCTGATGGATGCTCTGTACCTGACTCCTGCTGAGGCTGAGGTTGCCAAAACCAAGCTGCCACCAGGTGTTGCCATTCCACCATCTTCTGTTGGTTTCGATTTCGATGGTCCTGAACGTACTCTGAAGTTCTACATCCCATCTGTTCGTAAGGCATTGGCAACTGGTCAGGATGTCTCTGAACTCATGCTGAAGACTCTGCGTGGTTTGCAGCCACTTGGTTCTGAACTCGTTCCTGCTATGGATCTGATTGCCTCTTACCTGTCCACTCGTACCAACGACGCTATGCTTCCACTTGTTGGTATCGACTGTCTTGATCCACGTACTCACAAGAACGCTCGTGTCAAATGCTACCTGCACACCTCTTCCAACTCTTTCGCTGTTGTTCGTGATGTCCTGACTCTTGGTGGTCGTCTGTCTGATGATACCTCTCTGAAGCGTGTTGAGACTCTGAAATCCGTTTGGCCACTGCTGATCAACGAACTCGAAGGTCCACAGTCTGATGCTGCTACTATGGATGAGTCCTGGTCCAAGCCTGAACGTCTGAACCGTACTGGTTACTCTGGTATCCAGTACACCATCGAGATCACTCCTGGTCAGGCAATCCCTGATACCAAGATTTACGTTCCATTGTTCCAGTACACCGACTCTTCTGAGGTTGCTGAGCGTAACTTCGAGTCTGCACTGAAGAAGCTGGGTAACGAATGGGGTCTGTCTGGTAAGTACCGTTCTGTTATGCAGGAAATCTTCAAGGATGTCGAGAACTACGGTCAGACCTACGCATCCTTCTCCTACACCGAGGGTAAGGGTGTCTACACCACCTCCTACGTCGCTATGCCAATCAAGGATGAGGGTGGTGGTTCCCTGGCTGGTGACTTCGGTTTCCGTAACTAA

***tdiC***

ATGCACGCAGCTCTTGTTCCAACTTGGTCTTCTCCATGTCCAATCTATACTGAAATTCCAGATCCTGGTCCACCACCACCAGAACAGCTGCAACTGAAAGTCTTGGCTGTTGGTATCCCACGTGTTGTTCGTCTTCGTGCTCGTGGTATCCATCCTACTGCCAAGTCTGCCTCTCTTCCATACGATCCATCCATTGATGGTGTTGGTATCGACGAACAAACCGGTATCATGTACTACATCCTTCCACTCTCTGCCTCTTGTCTGGCTGAGAAAGTCAACGTTGACCGTGACAACCTTGTTCCACTTCAGCCTGGTGCACCAAAACCACAACCACGTAACGGTCCTGAGAACGGTTACGGTATCGCACTTGGTGACGCTGCTGATCATCGTGCTGAGACTCTGGACCCAATCGCAATTGCTGGTCTGGTCAATCCTGTCTCCTCTTCTTGGATGGCATTGCGTACTCGTGTTGATGGTGAGATCACTGGTAAGACTGTTCTGGTTCTTGGTGCTACTTCCAAGTCTGGTCGTGCTGCTGTTCTCGTTGCACGTTTCCTGGGTGCCAACAAAGTCATCGGTGTTGCACGTCGTGAGGAAGGTCTGCGTTCTGTCGAAGGTCTGGATGGTTGGGTCACTTCTGGTGACATGCTTCCTGGTGAGACTGGTGTTCGTTTCGCACTTCCTGACTGGGTTGGTCCTGTCCACATCGTCCTGGACTACGTTGGTGGTTCTGTTGCTGCTGGTGTTCTTGGTTCTGCTGAGATCGAGGAGGGTCGTGAGTTGCAATACGTCCAGGTTGGTAATCTGGCACTTGAGCTTGGTACTGGTGAGAAGCACATGTTCGAGACCTTGCCTGGTCATCTGATCTCTCGTAAGCCAATCTGCATTCGTGGTTCTGGTATGGGTTCTTTCTCTCGTCGTGATCTGGTTCGTGAGATGCCTGGTTTGGTTGCATTCCTTGCTCGTATGAAGGCACCATTCGGTATCGCATCTGCTCCAATGTGTGAGGTTGCATCCGTCTGGCAGGACGAGGACACCAAGGGTTCCCGTGTCGTCATCGTCCCTTAA

***tdiD***

ATGGGTTCTATTGGTGCTAACAATGCTGTTGCTGATCCAACTCCACTTTTCTCTTCCAGAGTTCAGAAATGGGAACCTGGTGCAATCCGTTCTCTTCTTCCACTGGAAGCCCTTCCTGGTATGATCTCCCTGGTTGCTGGTAAGCCATCTCCTGAGACCTTCCCAATCGCTGAGATCGCTATCTCTCTGAAGGACACTCCTGCTGGTACTGGTCGTATCGTTGTTGATGGTGATGAACTGAACCAGGCTCTGCAATACGGTCTTCCTCGTGGTAACGCACAGCTCATCCAGTGGTTCGAGTCTCTTCAGCGTTCTGTTCATGGTCTGGACGAGAATGGTGGTTGGTCTTGTTGCATCGGTAACGGTTCTCAGGAGCTGATCCATCGTGTCATCCAGGTCTTCACTGACCCTGGTGATCCAGTTCTGCTGGAAACTCCTGCATACCCTGGTGTTGCTGGTTTCCTTCGTGCTGATGGTCAGGAGCTGATCCCTGTCTACTCTGACGCTCAGGGTCTGAATCCTGCCTCTCTGGAACAGGCACTGTCTGAATGGCCTGGTGACTCTCCACGTCCTAAGGTCCTGTACACCACTCCAACTGGTTCCAACCCAACCGGTCAATCTTGCACTGAGTCTCGTAAGGCTGAGATCCTGCGTCTTGCCAAGCGTTTCAACTTCATCATCCTGGAAGACGATGCCTACTACTACCTGAACTACGGTGACGACAAACAGCGTGCACGTTCCTACCTTGCTCTGGAACGTGACGTCAACGGTGAGTCTGGTCGTGTCGTTCGTTTCGATTCTCTGTCCAAGATCGTCTCTCCTGGTATGCGTCTTGGTATCCTGACTGCACAAGCTGCTGTTGTTGACAAGGTTGTTCGTATCACTGAGAACATCAACCTTCAACCATCCTCCACCACCCAACTGCTTGCTCTGTCCCTTCTTCGTCACTGGGGTCAAGCTGGTTTCCTGAAGCACTGTGCTGAAGCTGCTGAGGTCTACCGTCGTCGTCGTGACGTCTTCGTCTCTGCTGCTGAGCGTCACCTTCAGGGTCGTGCTACTTGGGTCGTTCCAACTGCTGGTATGTTCGTCTGGCTGGAACTCAAGTTGCCACCTGAGATGGACTCCTTCGAGCTTCTGAAGTCCCAGGGTATGAAGAACGGTGTTCTCGCTATCCCTGGTGTTGCCTTCATGCCTGGTAACGAACAGACCTGCTACATCCGTGTCTCCTTCTCTCTCGTTCCTGAGCGTGATATGGATGAGGCATGTCGTCGTATCGCTGGTCTGGTCGATCGTTGCGCCTGCCACTCCTAA

***tdiE***

ATGGTTAACAGACCATACGCAAATATGAATGATCTTGCTAATGCTGTTGCTGAATCTGTTCAGAAATATCTGGACAACTCTGGTCAAGCCAGAAACGGTCTGTTCCAGAAAACCAACAACGGTGAACGTGACATCGAAACCGAGCGTCTGGCACTCCAGCACCAGCTCTTCCACCTGACTCTGGACGGTAAACTCCAGTTGTCTCCACTGCCTTCTCCAGTTCAATCCGTTCTGGACATCGCCACTGGTGACTCCACCTGGGTCCATGCCTTCGCTGAACAGAACCCATCTGCCTACATCGTTGCCAACGACCCATCTCCAACCTCCAAGATCCCACTTGGTCTGTCTGTCATCCCAGACGCTTCCGATGCCAACGAACCTTGGACCTACACCAGACAGTTCGACTTCGTCCACTGTAGACAGCACCATCGTCGTCTTGACGAACCACGTCTGTTCAAGCAGGCATTCTCTTTCTGACTCCTGGTGGTTGGCTGGAGATGCAGGAACTCTCCAACCCTGTCACCTCTGATGACGGTACTCTGTCCGAGAACAACCCACTGTCTCAGTGGGGTCGTCTTCTGATCGAGGCATCCAAGAAGATGAACCGTCCTGTCGATAACCCTGCCAAATACGAGACCTGGATGCGTGAGGCTGGTTTCGTCAACTGCCACACCGTTGCCTACAACTGGCCTACCAACCCTTGGCCTGCTGACGAGAAGGGTAAGACCTTGGGTTTGTGGAACCTGTACAACGTCTTGCAGCGTTTCGAAGAGTTCTCTGTCGCCTTGTTGGTCAAAGTCCTCGGTTGGGAGATGGACGATGCTAAGACCTTCTTGGGTAACGTCAAGGAGGAGCTGATGAACGAGGGTGTTCATGGTTACTGGCCTGTCTACGTTGTCTACGGTCAGAAGCCAGCTGCACCTGGTTCCGACGTCATCACCGACTCCGAGTAA

***sfp***

ATGAAAATCTACGGTATCTACATGGATCGTCCACTCTCACAGGAGGAGAACGAACGTTTCATGTCATTCATCTCACCTGAGAAGCGTGAGAAGTGTCGTCGTTTCTATCACAAGGAAGATGCACATCGTACTCTGCTGGGTGACGTCCTGGTTCGTTCTGTGATCTCACGTCAGTACCAGTTGGACAAGTCTGACATCCGTTTCTCAACTCAGGAGTATGGTAAGCCATGCATTCCTGACCTGCCTGATGCACACTTCAACATCTCTCACTCTGGACGTTGGGTGATCTGTGCATTCGACTCACAGCCAATCGGAATCGACATCGAGAAGACCAAGCCAATCTCACTTGAGATGCCTAAGCGTTTCTTCTCCAAGACTGAGTACTCTGACCTGCTTGCCAAGGACAAGGACGAGCAGACCGACTACTTCTACCATCTCTGGTCCATGAAGGAATCCTTCATCAAGCAAGGACGTCAACGTCTCATCGCATCTGCATAA

***ScCK***

ATGGTTCAAGAGTCACGTCCTGGTTCTGTTCGTTCATACTCTGTTGGCTACCAAGCACGTTCACGTTCATCATCACAACGTCGTCACTCCTTGACTCGTCAGCGTTCCTCACAACGTCTGATTCGTACCATCTCCATCGAGTCTGATGTGTCCAACATCACTGACGATGACGACTTGCGTGCTGTGAATGAGGGTGTTGCTGGTGTGCAACTGGACGTCTCTGAGACTGCCAACAAGGGTCCACGTCGTGCATCTGCCACTGATGTCACTGATTCCTTGGGTTCAACTTCATCTGAGTACATCGAGATTCCATTCGTGAAGGAGACCTTGGATGCATCCTTGCCATCCGACTACCTGAAGCAGGACATCTTGAATCTGATTCAGTCCCTGAAAATCTCCAAGTGGTACAACAACAAGAAGATCCAACCTGTGGCACAAGACATGAACCTGGTCAAAATCTCTGGTGCAATGACCAACGCAATCTTCAAGGTTGAGTACCCTAAGCTGCCATCCTTGCTGTTGCGTATCTACGGTCCTAACATCGACAACATCATTGACCGTGAGTATGAGTTGCAGATCCTGGCACGTCTGTCCTTGAAGAACATCGGTCCATCACTGTATGGCTGCTTCGTCAACGGTCGTTTCGAGCAGTTCCTGGAGAACTCCAAGACCTTGACCAAGGACGACATCCGTAACTGGAAGAACTCTCAACGTATCGCACGTCGTATGAAGGAGTTGCATGTTGGTGTTCCACTGTTGTCATCTGAACGTAAGAACGGTTCTGCCTGTTGGCAGAAGATCAACCAGTGGTTGCGTACCATCGAGAAGGTTGACCAGTGGGTTGGTGATCCTAAGAACATTGAGAACTCCTTGTTGTGTGAGAACTGGTCCAAGTTCATGGACATTGTGGATCGTTACCACAAGTGGCTGATCTCCCAAGAACAGGGTATCGAGCAAGTGAACAAGAATCTGATCTTCTGCCACAATGATGCACAGTATGGCAACTTGCTGTTCACTGCACCTGTCATGAACACTCCATCACTGTACACTGCACCATCCTCCACCTCACTGACCTCACAGTCATCATCCTTGTTCCCATCATCATCCAACGTGATTGTTGATGACATCATCAACCCACCTAAGCAGGAGCAATCACAAGACTCCAAGTTGGTGGTGATCGACTTCGAGTATGCTGGTGCCAACCCTGCTGCCTATGACCTTGCCAATCACCTGTCTGAGTGGATGTATGACTACAACAACGCCAAGGCACCACATCAGTGCCACGCTGATCGTTACCCTGACAAGGAACAGGTCTTGAACTTCTTGTACTCCTATGTCTCACATCTTCGTGGTGGTGCTAAGGAACCTATCGATGAAGAGGTTCAACGTCTGTACAAGTCCATCATTCAGTGGCGTCCAACTGTTCAACTCTTCTGGTCACTGTGGGCAATCCTTCAGTCTGGCAAGTTGGAGAAGAAGGAAGCATCCACTGCAATCACTCGTGAAGAGATCGGTCCTAACGGCAAGAAGTACATCATCAAGACTGAACCTGAGTCACCTGAAGAAGACTTCGTTGAGAACGACGATGAACCTGAAGCTGGTGTCTCCATCGACACCTTCGACTACATGGCATACGGTCGTGACAAGATCGCTGTCTTCTGGGGTGATCTGATTGGCCTTGGCATCATCACTGAAGAAGAGTGCAAGAACTTCTCCTCCTTCAAGTTCCTCGACACTTCCTACTTGTAA

***AtIPK***

GAACGAGCTGGAGAAGATTCACGATGAGAATCTGGAGGTTGTTGCCTGTCAGCTTCGTCAAGCTATGCTGGAGGGTTCTGCACCTTCCAAGGTCATCGGTATGGACTGGTCCAAGCGTCCTGGTTCATCTGAAATCTCTTGTGATGTGGATGACATCGGTGATCAGAAGTCCTCTGAGTTCTCCAAGTTCGTTGTTGTCCACGGTGCTGGTTCCTTCGGTCACTTCCAGGCATCACGTTCTGGTGTTCACAAGGGTGGTCTTGAGAAGCCTATCGTGAAGGCTGGCTTCGTTGCTACTCGTATCTCTGTCACCAACCTGAACCTTGAGATCGTTCGTGCACTTGCACGTGAGGGTATCCCTACCATCGGTATGTCTCCATTCTCATGTGGTTGGTCAACCTCCAAGCGTGATGTTGCATCTGCTGATCTTGCCACTGTTGCCAAGACCATCGACTCTGGCTTCGTTCCTGTTCTGCATGGTGATGCTGTCCTGGACAACATCCTTGGCTGCACCATCTTGTCTGGTGATGTGATCATCCGTCATCTTGCTGATCACTTGAAGCCTGAGTATGTTGTGTTCCTGACTGATGTCCTTGGTGTGTACGATCGTCCACCATCACCATCTGAACCAGATGCTGTGCTGCTGAAGGAGATCGCTGTTGGTGAAGATGGTTCATGGAAGGTTGTCAACCCACTGTTGGAGCACACTGACAAGAAGGTTGACTACTCTGTTGCAGCACACGACACTACTGGTGGTATGGAGACCAAAATCTCTGAAGCTGCCATGATTGCTAAGCTGGGTGTGGATGTGTACATCGTCAAGGCTGCCACCACTCACTCACAGCGTGCACTGAACGGTGACTTGCGTGACTCTGTTCCTGAAGACTGGCTTGGTACTATCATCCGTTTCTCCAAGTAA

**Table S2.** Primers used in this study

| **Name** | **Premer sequence (5’ to 3’)** |
| --- | --- |
| *tdiA*-F | TCCGTCAAGTGCATGGATGTC |
| *tdiA*-R | CAGACCACGCTCACGCAGGAC |
| *tdiB*-F | GCACTGAAGAAGCTGGGTAAC |
| *tdiB*-R | ACGGAAACCGAAGTCACCAGC |
| *tdiC*-F | ATCTCTCGTAAGCCAATCTGC |
| *tdiC*-R | GACGATGACGACACGGGAACC |
| *tdiD*-F | ATGTTCGTCTGGCTGGAACTC |
| *tdiD*-R | GCAACGATCGACCAGACCAGC |
| *tdiE*-F | AAGACCTTGGGTTTGTGGAAC |
| *tdiE*-R | GACGTCGGAACCAGGTGCAGC |
| *sfp*-F | TCTCACTCTGGACGTTGGGTG |
| *sfp*-R | TGCAGATGCGATGAGACGTTG |
| *ScCK-*F | GGTCCTAACGGCAAGAAGTAC |
| *ScCK-*R | GGAGGAGAAGTTCTTGCACTC |
| *AtIPK-*F | CCACTGTTGGAGCACACTGAC |
| *AtIPK-*R | GGAGAAACGGATGATAGTACC |

**Figure S1.** PCR amplified fragments using plasmids from engineered strains as templates. M, DL2000 marker. The plasmids were extracted when *OD*600 reached 0.6 after being cultured in LB medium.

**Figure S2.** LC-MS analysis of the BL-3 culture after chloroform extraction. (**A)** Total Ion Chromatogram (TIC) of the sample. **(B)** Mass spectrum of ochrindole D product (m/z (ES+): 423.2 [M+H]^+^). (**C)** Mass spectrum of terrequinone A product (m/z (ES+): 491.3 [M+H]^+^).
